# Dysfunctional cGMP Signaling Leads to Age-Related Retinal Vascular Alterations and Astrocyte Remodeling in Mice

**DOI:** 10.3390/ijms23063066

**Published:** 2022-03-12

**Authors:** Joseph M. Holden, Sara Al Hussein Al Awamlh, Louis-Philippe Croteau, Andrew M. Boal, Tonia S. Rex, Michael L. Risner, David J. Calkins, Lauren K. Wareham

**Affiliations:** Vanderbilt Eye Institute, Vanderbilt University Medical Center, Nashville, TN 37232, USA; joseph.m.holden@vanderbilt.edu (J.M.H.); sarah.a.al-awamlh@vumc.org (S.A.H.A.A.); louis-philippe.croteau@vumc.org (L.-P.C.); andrew.m.boal@vanderbilt.edu (A.M.B.); tonia.rex@vumc.org (T.S.R.); michael.l.risner@vumc.org (M.L.R.); david.j.calkins@vumc.org (D.J.C.)

**Keywords:** retinal vasculature, neurovascular unit, blood retinal barrier, astrocyte, connexin, gap junctions, neurodegeneration, retinal ganglion cell

## Abstract

The nitric oxide–guanylyl cyclase-1–cyclic guanylate monophosphate (NO–GC-1–cGMP) pathway is integral to the control of vascular tone and morphology. Mice lacking the alpha catalytic domain of guanylate cyclase (*GC1^−/−^*) develop retinal ganglion cell (RGC) degeneration with age, with only modest fluctuations in intraocular pressure (IOP). Increasing the bioavailability of cGMP in *GC1^−/−^* mice prevents neurodegeneration independently of IOP, suggesting alternative mechanisms of retinal neurodegeneration. In continuation to these studies, we explored the hypothesis that dysfunctional cGMP signaling leads to changes in the neurovascular unit that may contribute to RGC degeneration. We assessed retinal vasculature and astrocyte morphology in young and aged *GC1^−/−^* and wild type mice. *GC1^−/−^* mice exhibit increased peripheral retinal vessel dilation and shorter retinal vessel branching with increasing age compared to Wt mice. Astrocyte cell morphology is aberrant, and glial fibrillary acidic protein (GFAP) density is increased in young and aged *GC1^−/−^* mice, with areas of dense astrocyte matting around blood vessels. Our results suggest that proper cGMP signaling is essential to retinal vessel morphology with increasing age. Vascular changed are preceded by alterations in astrocyte morphology which may together contribute to retinal neurodegeneration and loss of visual acuity observed in *GC1^−/−^* mice.

## 1. Introduction

The nitric oxide–guanylyl cyclase-1–cyclic guanylate monophosphate (NO–GC1–cGMP) signaling pathway is integral to the control of vascular tone and morphology [1]. Recently, studies in humans and mice have implicated the NO–cGMP pathway in glaucoma-related retinal neurodegeneration. In humans, multiple genetic epidemiology studies have implicated aberrant NO–cGMP signaling in glaucoma disease pathology [2,3,4,5]. Genetic variants in the genes *GUCY1A3* and *GUCY1B3*, encoding the α1 and β1 subunit of GC1, are associated with female glaucoma cases that exhibit early paracentral visual field loss [3]. Furthermore, physiological studies have detected lower levels of NO and cGMP in the serum and aqueous humor of glaucoma patients [6,7].

Neurodegeneration of retina ganglion cells (RGCs) in glaucoma is due to an increase in sensitivity to intraocular pressure (IOP). Despite IOP-lowering regimens, however, patients still progress to vision loss and, thus, identification of other mechanisms by which RGCs may develop increased sensitivity to IOP is warranted. Our previous work identified cGMP signaling as an IOP-independent neuroprotective target for RGC neurodegeneration [8]. Mice deficient in the alpha catalytic domain of guanylate cyclase (*GC1^−/−^*) develop RGC degeneration with age, accompanied by modest elevations in IOP [3,8]. Increasing the bioavailability of cGMP prevents RGC loss in *GC1^−/−^* mice and in a mouse model of ocular hypertension (OHT; microbead occlusion model [9]), in an IOP-independent manner [8]. These previous findings indicate an alternative, IOP-independent pathological mechanism of GC1–cGMP signaling in RGC degeneration.

There is an increasing body of evidence that supports vascular pathogenic mechanisms in glaucoma, linking RGC degeneration to dysfunctional neurovascular physiology in the retina [10,11,12]. Vascular dysfunction in glaucoma is observed in both systemic and retinal vascular endothelium [13,14,15,16]. Normotensive glaucoma patients who develop RGC degeneration without elevations in IOP indicate that the pathophysiology of disease is, at least partially, independent of IOP mechanisms of degeneration [14]. However, it remains unclear whether vascular pathology precedes or is secondary to retinal degeneration itself.

In this study, we explored the hypothesis that dysfunctional cGMP signaling alters the retinal vascular unit in *GC1^−/−^* mice. We used immunohistochemical techniques to analyze temporal changes in retinal blood vessel structure and astrocyte morphology and distribution in *GC1^−/−^* mice and wild type (Wt) mice. Our results show that *GC1^−/−^* mice exhibit progressive retinal vessel dilation with increasing age. Additionally, astrocyte morphology and Connexin 43 (Cx43) expression in *GC1^−^^/−^* retinas is perturbed in young and aged animals. These results together indicate that dysfunctional GC1–cGMP signaling leads to abnormal retinal vasculature and progressive astrocyte disorganization with age. Alterations in the neurovascular unit may lead to compromised retinal vascular supply to RGCs that could exacerbate or trigger neurodegeneration and increase sensitivity to IOP.

## 2. Results

### 2.1. Peripheral Retinal Vessels Dilate with Age in GC1^−/−^ Mice

NO–cGMP signaling influences a number of vascular cell types to regulate endothelial permeability, cell growth and differentiation, and vasomotor tone [1]. Previously, vascular dysfunction was detected in aged *GC1^−/−^* mice in vivo [3]. However, measures of vascular morphology and complexity, as well as whether the vascular phenotype was progressive with age, were not assessed. To determine if dysfunctional cGMP signaling impacts retinal vasculature morphology with increasing age, we carried out immunohistological staining of whole-mount retina in young and aged mice. Age-matched 3- (young) and 15-month old (aged) Wt and *GC1^−/−^* retina were stained with isolectin-B_4_, a marker for endothelial cells [17]. Regions of interest (ROIs) from mid and peripheral retinas were selected so that almost the entire retina was analyzed without overlap; an outline of the process is shown in Supplemental Figure S1A. 

**Figure 1:**
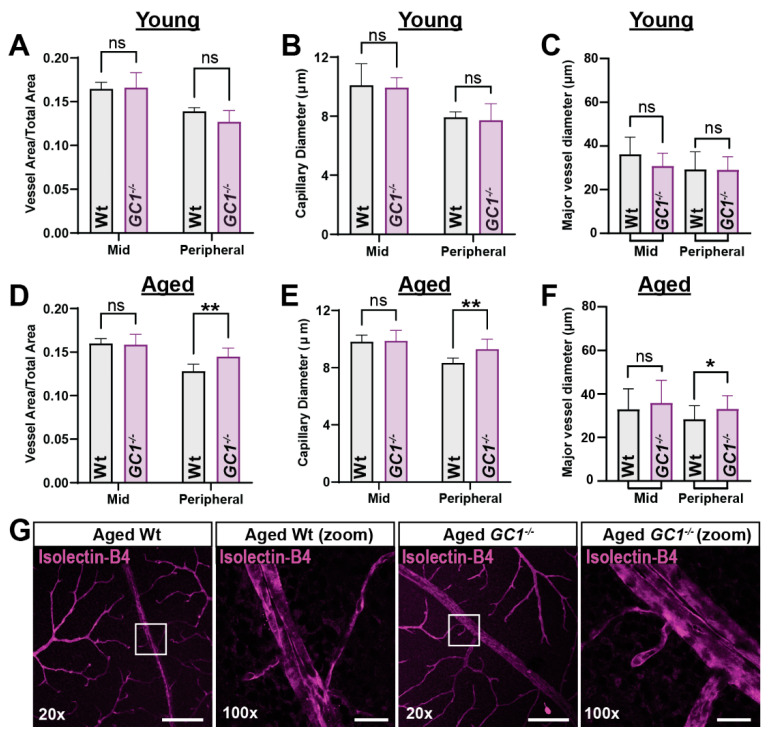
Aged *GC1^−/−^* mice exhibit dilated vessel morphology in peripheral retina compared with Wt mice. (**A**) Vessel density is not significantly changed in the mid or peripheral retina of young *GC1^−/−^* mice (*p* = 0.979 and 0.307, *n* = 4 mice per genotype group). (**B**) Capillary diameter is not significantly changed in the mid or peripheral retina of young *GC1^−/−^* mice (*p* = 0.970 and 0.949; *n* = 4 mice per genotype group). (**C**) Major vessel diameters are not significantly changed in young *GC1^−/−^* mice compared to Wt (*p* = 0.45 and *p* > 0.99; *n* = 10–16; measurements from 4 to 5 mice per genotype group at mid = 1300 µm and peripheral = 2350 µm from the optic nerve head). (**D**) Vessel density is significantly increased in the peripheral retina of aged *GC1^−/−^* mice (+12.8%; 0.128 ± 0.004 vs. 0.145 ± 0.004; ** *p* = 0.005, *n* = 7–9 mice per genotype group). (**E**) Capillary diameter is significantly increased in the peripheral retina of *GC1^−/−^* mice (+11.5%; 9.296 ± 0.293 µm vs. 8.339 ± 0.293 µm; ** *p* = 0.004; n = 7–9 mice per genotype group). (**F**) Major vessels are significantly more dilated in the peripheral retina of aged *GC1^−/−^* mice (28.360 ± 6.250 µm vs. 33.070 ± 6.100 µm; * *p* = 0.015; *n* = 19–26 measurements from *n* = 3 mice per genotype group at mid = 1300 µm and peripheral = 2350 µm from the optic nerve head). (**G**) Representative fluorescence images of whole-mount retina labeled with isolectin-B_4_ showing dilated vessels in aged Wt and *GC1^−/−^* mice. Scale bar = 200 µm at 20× and 25 µm at 100×. Data are presented as means ± S.D. Statistical tests performed were two-way ANOVA and Sidak’s multiple comparisons test. Each *n* represents one retina from one animal. ns: not significant.

Retinal vessels were first examined using REAVER [18], an automated pipeline for retinal vessel quantification. REAVER analyzes total blood vessel diameters, regardless of size; thus, most measurements reflect capillary diameters with some major vessel diameters included. We termed these data ‘capillary diameter’ (Figure 1B,E), with the caveat that some larger vessel data are included. We also measured major vessels manually (Figure 1C,F).

In young *GC1^−/−^* mice, total retinal vessel area and capillary diameter were unchanged in both mid and peripheral retina (Figure 1A; *p* = 0.979 and 0.307, and Figure 1B; *p* = 0.970 and 0.949). Major vessel diameter also remained unchanged between genotypes in mid and peripheral regions (Figure 1C; mid *p* = 0.45, peripheral *p* > 0.99). Next, we assessed retinal vasculature in aged mice. Overall, total vessel density was unchanged in mid retina, but vessel density increased in the periphery by 12.8% in *GC1^−/−^* mice compared with age-matched Wt mice (Figure 1D; 0.128 ± 0.004 vs. 0.145 ± 0.004; ** *p* = 0.005). There was a significant increase (11.5%) in capillary diameter in peripheral retina of *GC1^−/−^* mice compared with Wt (Figure 1E; 8.339 ± 0.293 µm vs. 9.296 ± 0.293 µm, ** *p* = 0.004). There was also a significant increase in major vessel diameter in peripheral retina of *GC1^−/−^* mice (Figure 1F, 28.360 ± 6.250 µm vs. 33.070 ± 6.100 µm; * *p* = 0.015). Representative confocal images of peripheral retinal vessels are shown in Figure 1G; 100× images demonstrate the increased vessel diameter phenotype. We also compared other vessel endpoints across ages within each genotype to assess changes with age (Supplementary Figure S2). We did observe a slight, non-significant decrease in overall vessel area in the periphery of wild type mice (Supplementary Figure S2A) and a non-significant increase in *GC1^−/−^* mice (Supplementary Figure S2B), which may have contributed to differences observed between genotypes. However, we saw a significant increase in capillary diameter in the periphery of *GC1^−/−^* mice between young and aged animals (Supplementary Figure S2D), which was not evident in wild type mice (Supplementary Figure S2E), suggesting that increased capillary diameters in the peripheral retina are indeed a genotype-specific trait.

### 2.2. Retinal Vessel Branches Are Shorter in Aged GC1^−/−^ Mice

To determine whether increased overall vessel density was due to increased complexity and branching of vessels, branch points were analyzed. A single branch point was determined by REAVER as the node at which multiple vessels diverge. There was no significant change in branching in both the mid and peripheral retina between wild type and *GC1^−/−^* mice (data not shown). These results together suggest that the increase in total vessel area observed peripherally in *GC1^−/−^* mice is primarily due to capillary vessel dilation and suggest that dysfunctional cGMP signaling leads to progressive vasodilation of retinal vessels with age compared to Wt mice.

**Figure 2:**
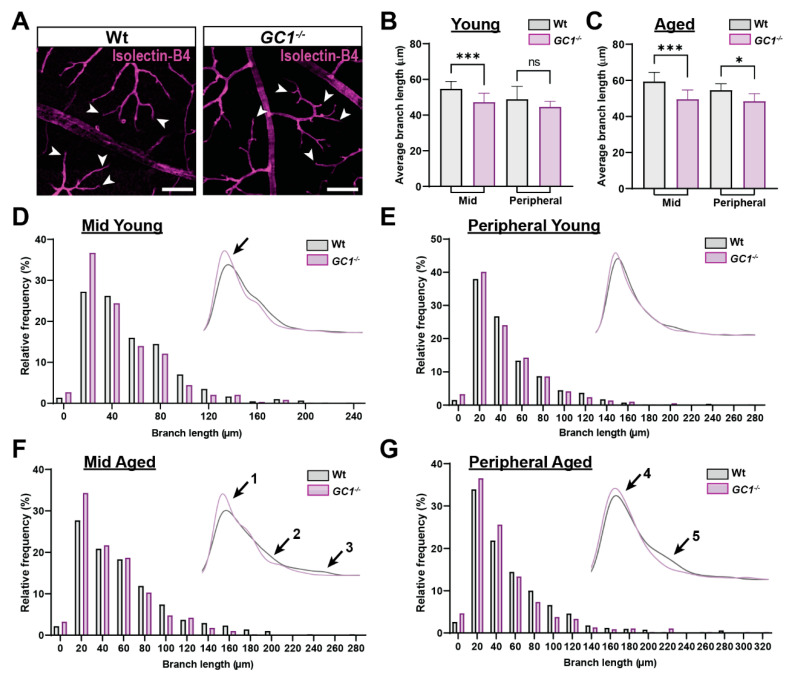
*GC1^−/−^* mice have an increased frequency of shorter capillary branches compared to Wt mice. (**A**) Representative fluorescent images of Wt and *GC1^−/−^* vessels stained with isolectin-B4. White arrows give example of a selection of terminal vessels, scale bar = 100 µm. (**B**) In mid retina of young mice, average branch length was significantly shorter in *GC1^−/−^* mice compared with age-matched Wt mice. No significant difference was observed in the peripheral retina between genotypes (*n* = 6 mice per genotype group; *** *p* = 0.0002 and *p* = 0.46). (**C**) In mid retina of aged mice, average branch length was significantly shorter in *GC1^−/−^* mice compared with age-matched Wt mice (*n* = 3 mice per genotype group; *** *p* = 0.0051). Average branch length in the peripheral retina of aged *GC1^−/−^* mice was significantly shorter than in aged Wt mice (*n* = 3 mice per genotype group; * *p* = 0.022). (**D**) Histogram showing normalized frequency of branch lengths in the mid retina of young Wt and *GC1^−/−^* mice; inset: Kernel density estimation (KDE) plot. (**E**) Histogram showing normalized frequency of branch lengths in the peripheral retina of young Wt and *GC1^−/−^* mice; inset: KDE plot. (**F**) Histogram showing normalized frequency of branch lengths in the mid retina of aged Wt and *GC1^−/−^* mice; inset: KDE plot. There is an increase in the frequency of shorter vessel lengths (arrow 1) and a decreased frequency of longer vessel lengths in *GC1^−/−^* mice (arrows 2 and 3). (**G**) Histogram showing normalized frequency of branch lengths in the peripheral retina of aged Wt and *GC1^−/−^* mice; inset: KDE plot. The frequency of shorter branch lengths was increased in *GC1^−/−^* mice (arrow 4) compared with Wt and a lower frequency of longer branch lengths was observed in *GC1^−/−^* mice (arrow 5). Data are presented as means ± S.D. Statistical analyses carried out were Kruskal–Wallis one-way ANOVA. ns: not significant.

Although the number of total vessel branch points did not change between Wt and *GC1^−/−^* mice, visual analysis of isolectin-B_4_-labeled whole-mount retinas raised the possibility that terminal blood vessel branches in aged *GC1^−/−^* mice had reduced length compared to age-matched Wt controls; example images of tertiary branches are shown in Figure 2A (white arrow heads). To quantify these changes, we manually measured tertiary vessel lengths in Wt and *GC1^−/−^* retinae, in both young and aged animals. In young mice, average branch lengths in *GC1^−/−^* mice were shorter compared with Wt mice in mid retina (Figure 2B; *** *p* = 0.0002), but no significant difference was observed in peripheral retina between genotypes. In aged mice, however, significantly shorter vessel branches were found in the *GC1^−/−^* retinae at both mid and peripheral regions (Figure 2C mid retina ** *p* = 0.0051, peripheral retina * *p* = 0.022). No significant changes were observed for either genotype with increasing age (Supplemental Figure S3).

To get a better view of the distribution of vessel lengths across the retina in both genotypes, we plotted frequency histograms and kernel density estimation (KDE) plots (Figure 2D–G). In mid retina of young *GC1^−/−^* mice, there is a slight shift towards shorter branch lengths compared to Wt (Figure 2D, inset; black arrow). In the peripheral retina, this shift is not observed, which is indicative of no change in vessel lengths (Figure 2E). In aged mice, vessel branches were shorter in both mid and peripheral retina of *GC1^−/−^* mice (Figure 2F,G). In mid retina, the most prominent difference in vessel length frequency occurred in bins where branch lengths were shorter than approximately 25–30 µm. In these bins, *GC1^−/−^* animals had dramatically increased vessel frequency (Figure 2F; inset arrow 1). Additionally, at longer vessel lengths, the KDE plot lines diverge, and the Wt animals dominate the longer length vessel frequencies (Figure 2F; inset arrows 2 and 3). A similar trend was seen in the frequency distributions in the periphery (Figure 2G). A slight shift towards *GC1^−/−^* mice having a higher frequency of branch lengths measuring 20–40 µm (Figure 2G, inset; arrow 4), paired with Wt mice having a higher frequency in longer branch lengths (Figure 2G, inset: arrow 5), indicates overall decreased branch lengths across the retina of aged *GC1^−/−^* mice. Taken together, these data indicate that vessel lengths in *GC1^−/−^* mice become progressively shorter with age compared with Wt mice.

### 2.3. Dysfunctional cGMP Signaling Alters Astrocyte Morphology

**Figure 3:**
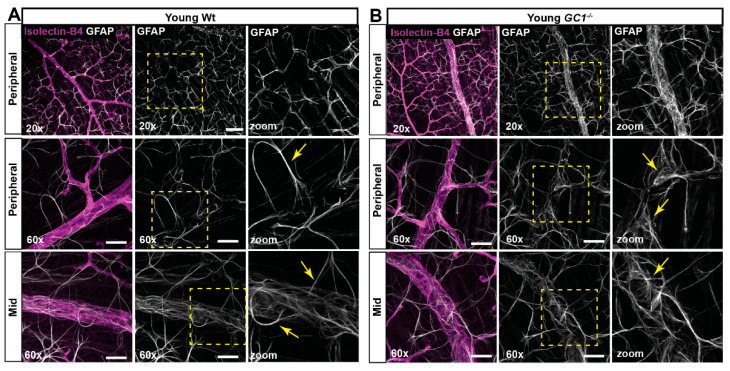
Astrocyte morphology in young Wt and *GC1^−/−^* retina. (**A**) Representative confocal micrographs of astrocytes (GFAP; white) and blood vessels (isolectin B4; magenta) in peripheral and mid retina of young Wt mice. Astrocyte processes are long and striated (yellow arrows) with end feet wrapping the vessels. (**B**) Representative confocal micrographs of astrocytes (GFAP; white) and blood vessels (isolectin B4; magenta) in peripheral and mid retina of young *GC1^−/−^* mice. Astrocyte processes are long, but end feet are increasingly frayed proximal to vessels (yellow arrows). Scale bars at 20× = 100 µm and at 60× = 40 µm.

**Figure 4:**
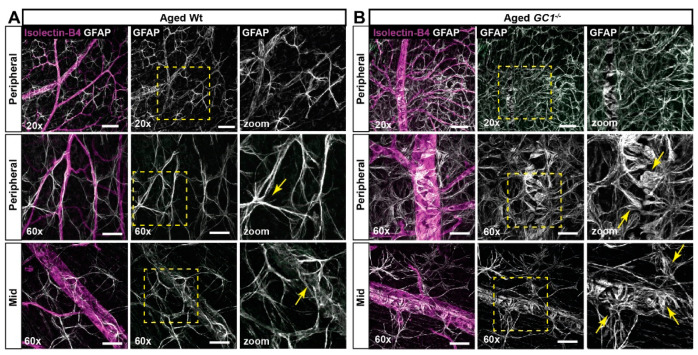
Astrocyte morphology in aged Wt and *GC1^−/−^* retina. (**A**) Representative confocal micrographs of astrocytes (GFAP; white) and blood vessels (isolectin B4; magenta) in peripheral and mid retina of aged Wt mice. Astrocyte processes are increasingly striated and frayed (yellow arrows) in proximity to vessels compared with young mice. (**B**) Representative confocal micrographs of astrocytes (GFAP; white) and blood vessels (isolectin B4; magenta) in peripheral and mid retina of aged *GC1^−/−^* mice. Dense patches of matted astrocytes are observed in proximity to blood vessels at the periphery. End feet appear increasingly bulbous and frayed (yellow arrows) compared to young *GC1^−/−^* animals and Wt mice. Scale bars at 20× = 100 µm and at 60× = 40 µm.

Astrocytes are an integral component of the neurovascular unit, regulating and maintaining the BRB [19,20]. Since we observe a retinal vascular phenotype with age in *GC1^−/−^* mice, we investigated whether astrocytes were morphologically changed. Using confocal microscopy, we visualized GFAP-labelled astrocytes and retinal vessels in young (Figure 3) and aged (Figure 4) mice. In young Wt animals, astrocytes covered the retina in a uniform web-like pattern, forming extensive networks between vessels (Figure 3A). Astrocyte processes appeared thin, with fan-like end feet making fine connections to retinal blood vessels (Figure 3A; yellow arrows). In young *GC1^−/−^* mice, astrocytes also covered the retina, but upon close inspection, there were subtle differences in the organization of astrocyte processes and end feet connecting to vessels (Figure 3B). Processes appeared to be more tortuous at the point of vessel interaction, and end feet processes were slightly thicker and more striated (Figure 3B; yellow arrows).

There was a striking difference in astrocyte morphology between age groups in both genotypes. In aged Wt mice, astrocyte processes appeared thicker, more striated, and more tortuous than young Wt mice at the vessel interaction point (Figure 4A; yellow arrows). In aged *GC1^−/−^* mice, the phenotype was more severe—we observed a less uniform spread of GFAP astrocyte staining, with large clusters of severely matted astrocytes primarily around major blood vessels that was absent in young *GC1^−/−^* mice (Figure 4B). Closer images of astrocyte end feet in matted patches of astrocytes showed enlarged processes with bulbous end feet surrounding blood vessels (Figure 4B; yellow arrows). A similar phenotype was observed elsewhere in the retina where astrocytes were not as densely packed, indicating a progressive remodeling of astrocytes in *GC1^−/−^* mice with age.

### 2.4. Dysfunctional cGMP Signaling and Increasing Age Alter Astrocyte Density and Complexity

**Figure 5:**
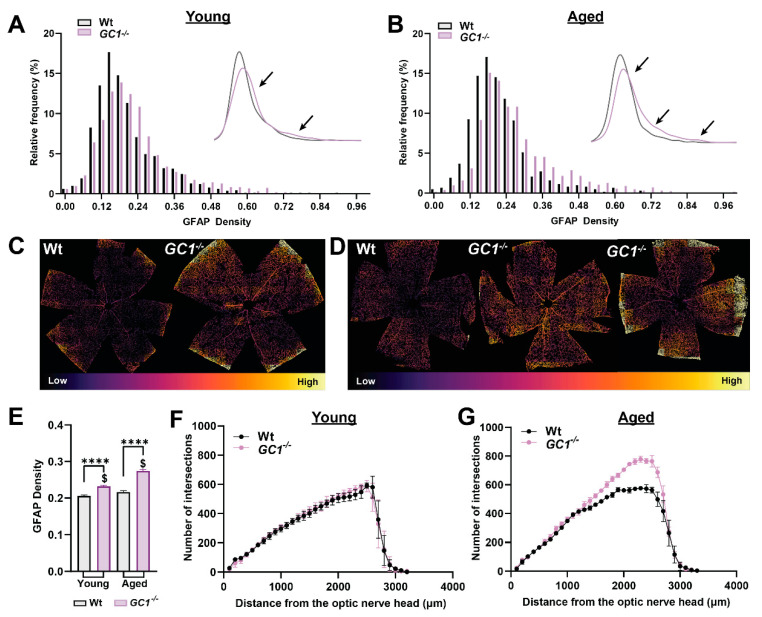
Astrocyte density is increased in *GC1^−/−^* retina. (**A**) Histogram plots of GFAP density across whole retinas of young Wt and *GC1^−/−^* animals (*n* = 8 Wt, *n* = 10 *GC1^−/−^*). The subplot shows a kernel density estimation plot for clarity; arrows signify differences in distributions between genotypes. (**B**) Histogram plots of GFAP density across whole retinas of aged Wt and *GC1^−/−^* animals (*n* = 3 Wt, *n* = 4 *GC1^−/−^*). The subplot shows a kernel density estimation plot for clarity; arrows signify differences in distributions between genotypes. (**C**) Representative retinas of young Wt and *GC1^−/−^* mice recolored according to relative GFAP density. (**D**) Representative retinas of aged Wt and *GC1^−/−^* mice pseudo-colored according to relative GFAP density; arrows point to areas of increased density. (**E**) Bar plot comparing GFAP densities between all groups. Data plotted as mean ± SEM. (**** *p* < 0.0001 and $ *p* < 0.0001). (**F**) Scholl analysis of binarized GFAP in young mice and (**G**) aged mice. Statistical analysis carried out was Kruskal–Wallis one-way ANOVA.

To quantitate how astrocyte morphology changes with age and genotype, we developed a script to assess GFAP density changes across the entire retina. GFAP is commonly used as a marker for astrocytes due to its prominent staining of cell soma and some processes [21]. IHC-dependent analysis of cell morphology can be biased due to sampling methods, so we sought to develop a method that assesses total GFAP density across complete retinal samples, removing sampling bias. We first plotted normalized frequency histograms for GFAP density in young (Figure 5A) and aged mice (Figure 5B). In young Wt mice, the frequency distribution of GFAP density is sharper than in *GC1^−/−^*, which is slightly shifted towards the higher frequencies (Figure 5A). A KDE plot was used to illustrate the shifts in GFAP density distribution (Figure 5A, inset; black arrows).

In aged mice, significant shifts towards higher GFAP density were only apparent in *GC1^−/−^* mice (Figure 5B). The higher GFAP densities in *GC1^−/−^* mice appeared in conjunction with fewer patches of lower density GFAP areas (Figure 5B, KDE plot arrows). Figure 5C,D provide examples of pseudo-colored GFAP density plots for whole retina in Wt and *GC1^−/−^* mice; areas of higher density GFAP appear as warmer colors, compared with cooler colored low-density areas. In young mice, *GC1^−/−^* experience a higher frequency of dense GFAP staining than Wt mice. Similarly, aged *GC1*^−/−^ mice exhibit large patches of denser GFAP staining, particularly evident in the peripheral retina compared with aged Wt mice (Figure 5D). When average retinal GFAP densities were plotted, GFAP density was significantly increased between genotypes in young and aged mice (Figure 5E, **** *p* < 0.0001). GFAP density was not significantly increased with increasing age in Wt mice (*p* = 0.246) but was significantly increased with age in *GC1^−/−^* mice (Figure 5E, $ *p* < 0.0001).

The observed increase in areas of high GFAP density between Wt and *GC1^−/−^* mice and the progressive increase in GFAP density with age in *GC1^−/−^* mice could arise due to multiple factors in isolation or consortium. For example, an increase in astrocyte process thickness, complexity, total cell number, as well as astrocyte migration could all function to increase focal astrocyte density in the retina. To probe the mechanism behind the increase in density that we observed between age groups and genotypes, we carried out Sholl analyses on entire whole-mount retinas labeled with GFAP. Sholl analysis is typically used to assess neuronal dendritic arbor complexity [22], but has been extended for use in retinal vasculature [23] and in astrocytes [24]. We used Sholl analysis to assess the astrocyte network across the retina in young (Figure 5F) and aged (Figure 5G) mice. Increases in the number of intersections could be indicative of either increased astrocyte complexity (number of astrocyte processes) or an increase in the number of astrocytes themselves. Additionally, if astrocytes migrate within the retina, increases in intersections in one region would be accompanied by decreased intersections in another. Interestingly, no differences in the Sholl distributions were found between young Wt and *GC1^−/−^* mice, suggesting that the primary contribution to shifts in GFAP density was not due to either increases in the number of astrocyte processes or the total number of astrocytes in isolation. Rather, the primary driver for the observed increase in GFAP density between genotypes is likely astrocyte process thickening. In aged mice, however, there was a large difference in the Sholl distribution towards the peripheral retina (Figure 5G, 1000–3000 µm from the optic nerve head), with *GC1^−/−^* mice showing an increased number of intersections compared to Wt. The fact that the distributions do not differ up to 1000μm from the optic nerve head suggests that the increase in density is not due to astrocyte migration from this region. Our data together suggest that the increase in astrocyte density in aged *GC1^−/−^* mice is due to a combination of factors, including increased process complexity and process thickening. These results demonstrate that both age and dysfunctional cGMP signaling alter the astrocyte network in the retina.

### 2.5. Astrocytic Cx43 Decreases with Age in the Retina

**Figure 6:**
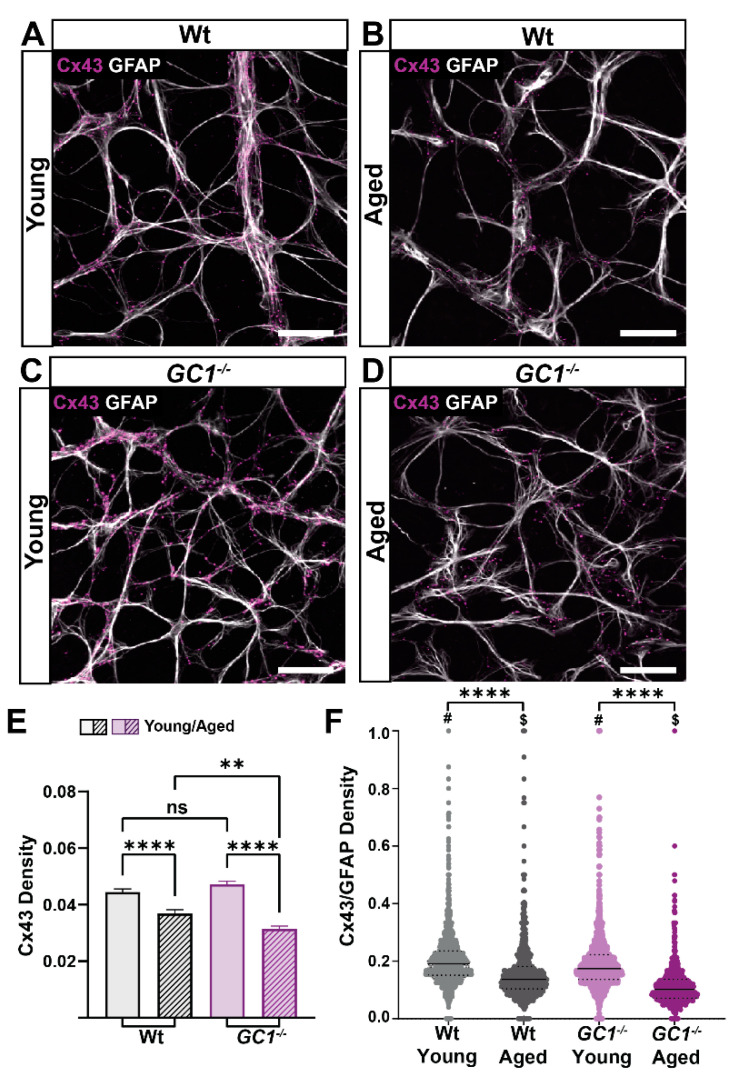
Connexin 43 (Cx43) density decreases significantly with age in Wt and *GC1^−/−^* mice. (**A**) Representative confocal micrographs of astrocytes (GFAP; white) and Cx43 (magenta) in retina of young Wt mice. (**B**) Representative confocal micrographs of astrocytes (GFAP; white) and Cx43 (magenta) in retina of aged Wt mice. (**C**) Representative confocal micrographs of astrocytes (GFAP; white) and Cx43 (magenta) in retina of young *GC1^−/−^* mice. (**D**) Representative confocal micrographs of astrocytes (GFAP; white) and Cx43 (magenta) in retina of aged *GC1^−/−^* mice. Scale bar = 40 µm. (**E**) Cx43 density across entire retinae of young and aged Wt and *GC1^−/−^* mice. Cx43 significantly decreases with age in Wt mice (*n* = 8 Wt, *n* = 10 *GC1^−/−^*; **** *p* < 0.0001) and significantly decreases with age in *GC1^−/−^* mice (*n* = 3 Wt, *n* = 4 *GC1^−/−^;* **** *p* < 0.0001). There is a significant difference between aged Wt and *GC1^−/−^* mice (*n* = 8 Wt and *n* = 4 *GC1^−/−^*; ** *p* = 0.005) (**F**) The ratio of Cx43 to GFAP density for each quadrant analyzed shows a significant difference between young and aged Wt mice (**** *p* < 0.0001) and young and aged *GC1^−/−^* mice (**** *p* < 0.0001). There is also a significant difference between genotypes in young (# *p* < 0.0001) and aged ($ < 0.0001) mice. All data expressed as means ± S.E.M. Statistical analyses carried out were Kruskall–Wallis one-way ANOVA. ns: not significant.

Astrocyte networks rely heavily on gap junction and hemichannel proteins, such as the connexin family of proteins, for inter-cell communication [25]. The most abundantly expressed connexin in astrocytes is connexin-43 (Cx43) [26]. Since we saw an increased density of GFAP in young and old *GC1^−/−^* mice, we investigated whether this upregulation was associated with changes in Cx43 density. Astrocyte expression of Cx43 was evident along processes and in cell soma of young mice (Figure 6A). In aged Wt mice, the Cx43 expression appeared lower, but in a similar distribution to young mice (Figure 6B). Similarly, Cx43 expression was evident in young *GC1^−/−^* mice (Figure 6C) but appeared decreased in aged *GC1^−/−^* mice (Figure 6D). Using the same script developed to assess GFAP density, we quantitated density of Cx43 in whole retinas of the same animals in which GFAP density was quantified (Figure 6E). Interestingly, Cx43 density significantly decreased with increasing age in Wt mice (**** *p* < 0.0001). In *GC1^−/−^* mice, the decrease was also pronounced with age (**** *p* < 0.0001). There were no significant differences in Cx43 density between genotypes in young mice, but a significant difference was observed between aged Wt and *GC1^−/−^* mice (** *p* = 0.005). To determine if the Cx43 decrease was associated with changes in GFAP density, we first determined whether Cx43 staining was colocalized to GFAP staining by determining Mander’s coefficients for the GFAP and Cx43 channels of confocal images of the retina. Cx43 staining was colocalized to GFAP in both young and aged retinas (data not shown; Wt M1 = 0.732 ± 0.03, *GC1^−/−^* M1 = 0.769 ± 0.03), with no significant difference observed between genotypes (data not shown; *p* = 0.19, Student’s *t*-test). Next, we expressed Cx43 density as a ratio of GFAP density in each tile analyzed. There was a significant difference in Wt and *GC1^−/−^* mice with age (Figure 6F; **** *p* < 0.0001). Furthermore, there was a significant decrease between genotypes at each age point (Figure 6F; Wt #*p* < 0.0001, *GC1^−/−^* $ *p* < 0.0001). Despite an increase in GFAP density between young and aged *GC1^−/−^* mice, Cx43 expression was not correspondingly increased. These data together indicate that increased age reduces Cx43 density within retinal astrocytes. Mice lacking functional GC1 experience an apparent greater loss with age in Cx43 despite increased GFAP.

### 2.6. Visual Acuity Is Decreased with Age in GC1^−/−^ Mice

**Figure 7:**
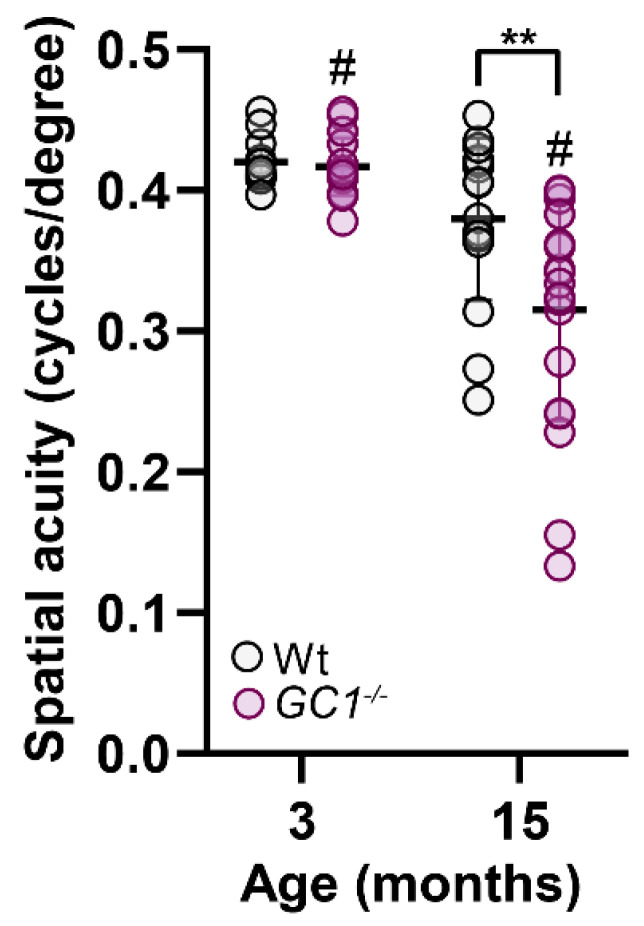
Visual acuity is decreased with age in *GC1^−/−^* mice. Visual acuity in Wt mice does not significantly decline with age (3 m; *n* = 6 and 15 m; *n* = 8), however, *GC1^−/−^* visual acuity significantly declines (3 m vs. 15 m, *n* = 10 and *n* = 8 # *p* < 0.0001; Kruskal–Wallis one-Way ANOVA). *GC1^−/−^* have similar acuity to Wt mice at 3 months (*n* = 10), which declines significantly at 15 months compared to Wt mice (*n* = 10; ** *p* = 0.02; Kruskal–Wallis one-Way ANOVA). Data points represent independent acuity readings in each naïve eye and are plotted as means ± S.D.

It is well documented that *GC1^−/−^* mice lose retinal ganglion cells with age [3,8], and that increasing the bioavailability of cGMP prevents degeneration [8]. Since our *GC1^−/−^* mouse is not a conditional knockout and GC1 is implicated in photoreceptor physiology, we assessed whether changes in visual acuity were evident in young and aged animals (Figure 7). No visual acuity deficits were evident in young animals between genotypes. Although Wt visual acuity was non-significantly reduced, *GC1^−/−^* mice had significantly reduced visual acuity at 15 months of age compared to Wt mice and younger *GC1^−/−^* mice (Figure 7). These data support previous findings of thinning of the nerve fiber layer and RGC loss with age in *GC1^−/−^* mice [3,8].

## 3. Discussion

Previous work has implicated dysfunctional NO–cGMP signaling in the pathophysiology of human glaucoma [2,3,4,5,6] and age-related RGC degeneration in *GC1^−/−^* mice [3,8,27,28]. However, the exact mechanisms that underly this pathology are not well understood [3]. We previously showed that tadalafil, a phosphodiesterase type 5 (PDE5) inhibitor, increased cGMP levels and prevented age-related degeneration of RGCs in *GC1^−/−^* mice and in the microbead occlusion model of ocular hypertension [9]. The neuroprotection afforded by elevated cGMP occurred in an IOP-independent manner, suggesting alternative mechanisms of RGC degeneration that warranted further investigation. Since NO–cGMP signaling is integral in the regulation of vascular tone, we sought to determine whether dysfunctional cGMP signaling impacts retinal vasculature and cells of the neurovascular unit to the extent that it may enhance susceptibility of RGCs to degeneration.

Our first significant finding is that dysfunctional cGMP signaling leads to changes in peripheral vascular morphology with age. Blood vessels in the peripheral retina of *GC1^−/−^* mice are dilated and mice exhibit shorter retinal capillary branching compared with Wt mice (Figure 1 and Figure 2). cGMP usually leads to vasodilation, so it is perhaps, at first, surprising that aged *GC1^−/−^* mice would experience dilation of vessels, rather than constriction. However, vasodilation of blood vessels is mediated by a delicate balance between multiple signaling pathways, including NO signaling and endothelin-1 (ET-1) [29]. The local absence of one pathway can disrupt the balance in signaling, leading to a disruption in vessel tone.

Our next significant finding was that *GC1^−/−^* -associated changes in astrocyte morphology preceded retinal vascular breakdown. We analyzed total astrocytes in each retina, minimizing sampling bias. We found that young *GC1^−/−^* mice had GFAP-labelled processes that were more frayed and less organized than Wt mice. In both young and aged *GC1^−/−^* mice, GFAP density was significantly higher than Wt controls, suggesting that astrocyte coverage in the retina was increased with dysfunctional cGMP signaling. In *GC1^−/−^* mice, an increase in GFAP density was observed with increasing age, which was not observed in Wt mice. The increased GFAP density observed in aged *GC1^−/−^* mice was driven by increased GFAP complexity in the peripheral retina. Since the vascular phenotype observed in *GC1^−/−^* mice was predominantly found in the peripheral retina, and we observed increased astrocyte matting surrounding blood vessels, it is plausible that gross astrocytic changes with age were driven by retinal vessel changes. Interestingly, the astrocyte morphology changes we observed are remarkably similar to what has been observed in murine retinal astrocytes following induced retinal detachment [30], in murine models of glaucoma [31,32] and retinitis pigmentosa [33]. Changes in glial density are also associated with human glaucomatous retinas [34] and in the aging retina [35]. This broaches the question whether pathological programs that promote early astrocytic morphological changes are shared between neurodegenerative retinal diseases, or elsewhere in the CNS.

An important point to note is that our study uses GFAP as an immunohistological label for astrocytes, however, GFAP only labels astrocyte soma and some processes and is therefore not a reliable indicator of cell reactivity status [23,36,37,38]. The distinct roles of astrocyte subtypes and reactivity in neurodegenerative disease has been recently debated in the literature [23]. In animal and human glaucoma studies, reactive astrocytes are observed early in disease progression, eventually resulting in glial scar formation in conjunction with RGC loss [39,40,41,42,43]. Reactive astrocytes contribute to neurodegeneration, and while we do observe increased GFAP labeling in *GC1^−/−^* mice, we cannot make a definitive claim on reactivity status of the cells without additional labeling and characterization.

An interesting finding is that retinal astrocyte Cx43 density was reduced with age in both genotypes and a greater decrease observed in *GC1^−/−^* compared to aged Wt mice. Connexins have rapid turnover rates and have been shown in other tissues, such as bone, to decrease in expression with age [44]. Additionally, it is known that cyclic nucleotides are involved in dynamic regulation of gap junction permeability and turnover [45]. It is possible that by disrupting the cGMP signaling pathway, the process of gap junction turnover and renewal is affected, leading to more greatly reduced levels in the *GC1^−/−^* mice compared to Wt with age. Furthermore, Cx43 mediates the redistribution of metabolic resources in response to retinal injury [46] and has a role in the spread of pro-survival and pro-apoptotic molecules in response to injury [47]. Reductions in Cx43 through blockade have been associated with neuroprotective responses [48], and therefore reduced connexin expression could be useful in slowing the spread of degenerative factors.

Our results suggest that disruption of the neurovascular unit precedes the age that RGC degeneration is observed in *GC1^−/−^* mice, and that breakdown of the neurovascular unit may contribute to or coincide with RGC degeneration and loss of visual acuity. Astrocytes, as part of the neurovascular unit, are essential in the maintenance of the blood retinal barrier; changes to cells in unit risk disruption of neurovascular coupling, which impacts RGC function [12]. We observed considerable changes in astrocyte morphology in young *GC1^−/−^* animals. Astrocytes express GC1 [49] and cGMP signaling directly impacts astrocyte cytoskeleton dynamics and motility [50]. Interestingly, GC1 expression and activity is reduced in reactive brain astrocytes of Alzheimer’s patients and in other neurodegenerative diseases [51,52]. Furthermore, a complex interplay between pro-inflammatory cytokines and GC1 expression has been shown [53]. Reduced GC1 expression in aging rodent and human brains [54,55] could be related to increased inflammation and the elevated risk for neurodegeneration that occurs with age. It is therefore possible that a lack of functional GC1 in mice leads to dysfunctional, pro-reactive retinal astrocytes with age, however, whether cGMP signaling is directly impacting astrocyte physiology will require further studies.

Astrocytes are just one cellular component of a plethora of cell types that comprise the neurovascular unit, which includes vascular cells (vascular smooth muscle cells, pericytes, and endothelial cells), glial cells (microglia, muller glia and oligodendrocytes), and neurons [12]. In addition to astrocytes, cGMP signaling has been implicated in the physiology and signaling of neurons, endothelial, and microglial cells [56]. It is possible that dysfunctional cGMP signaling is impacting other cell types in the neurovascular unit, which may have a contributing effect on retinal vasculature. Future studies will focus on exploring the role of cGMP signaling in other cell types to further characterize the vascular phenotype observed in *GC1^−/−^* mice. In conclusion, our findings support an integral role for functional cGMP signaling in the maintenance of the retinal vasculature and astrocytic cells of the neurovascular unit. Disruption in cGMP signaling leads to gross astrocyte remodeling and retinal vascular deterioration with age that may promote or trigger neurodegeneration of RGCs.

## 4. Materials and Methods

### 4.1. Animals

All animal studies were conducted in accordance with the National Institutes of Health (NIH) Guide for the Care and Use of Laboratory Animals and were approved by the IACUC at Vanderbilt University Medical Center. For the studies described, age-matched female wild type Sv/129S6 (Wt) and female *GC1^−/−^* mice on an Sv/129S6 background were bred and housed at the Vanderbilt University Medical Center. Young mice were aged 12–14 weeks and aged mice were 65–72 weeks. Mice were maintained on a 12 h light/dark cycle with ad libitum access to standard mouse chow and water. All mice were euthanized with 10 mg intraperitoneal pentobarbital injection. Observers, masked to animal genotype, experimental group, and diet, performed all data acquisition and analyses described.

### 4.2. Tissue Preparation

Following transcardial perfusion of 4% paraformaldehyde (PFA), eyes were punctured with a needle at the limbus and the cornea removed using spring scissors. The lens was removed and discarded, and the eyes were then post-fixed for 1.5 h. in 4% PFA with shaking. Retinas were dissected and effective removal of vitreous was assessed by staining the tissue with 1% toluidine blue. The retinas were stored in 1× phosphate buffered saline (PBS) + 0.02% sodium azide until used.

### 4.3. Immunohistochemistry, Imaging and Analysis

Perfused retinas were immunolabeled using the following antibodies and conjugates: GFAP (goat 1:500; Abcam, Cambridge, UK, ab53554), isolectin GS-IB_4_ biotin-XX conjugate (1:200; Invitrogen, Waltham, MA, USA, I21414), and connexin-43 (Rabbit 1:200; Cell Signaling Technology 3512S). Briefly, whole-mount retinas were blocked for 2 h at room temperature in 5% donkey serum in 1 × PBS with 0.1% triton. Primary antibodies were incubated overnight in 3% donkey serum 1 × PBS with 0.1% triton at 4 °C for 3–5 days with gentle rocking. Secondary antibodies from Jackson ImmunoResearch Laboratories Inc. conjugated to fluorescent fluorophores were incubated for 2 h at room temperature, also with gently rocking. Retinal whole-mount images were acquired using an Olympus FV-1000 inverted confocal microscope and a Nikon Ni Eclipse fluorescence microscope. For comparative quantification of immunohistochemistry (IHC), analyses were carried out on samples stained in the same experiment and images taken using the same imaging parameters. Total integrated density of each channel was quantified using Fiji software [57].

### 4.4. REAVER Analysis

Retinal vasculature was assessed using the open-source software REAVER [18], and details of the pipeline are shown in Supplemental Figure S1. Fluorescence images of the superficial ganglion cell layer were used in the analysis. In total, 1.44 mm^2^ regions of interest were chosen from proximal and distal regions of each retinal petal, covering the majority of the tissue from the optic nerve head to the edge of the retina. Using ImageJ, these regions of interest were processed prior to REAVER analysis. The background was subtracted using a rolling ball radius of 50 pixels followed by thresholding to remove background speckling while retaining full vasculature form. Intra-vessel differences in labeling intensity led to speckled coverage of some of the larger vessels, which interferes with the REAVER algorithm. This was fixed by using the ImageJ brush tool to fill those areas. Processed images were loaded into REAVER and subjected to the algorithm. 

### 4.5. Manual Vessel Diameter Measurements

Using fluorescence images of a whole-mount retina stained with isolectin-B_4_, circles of radius 1300, 2000, and 2350 µm from the center of the ONH were rendered in ImageJ, and any intersecting arteriole had its diameter measured at that location.

### 4.6. Manual Vessel Branching Measurements

Using whole-mount retinas labeled with the vasculature marker isolectin-B_4_, 1.44 mm^2^ regions of interest were rendered and subjected to manual vessel length measurements using ImageJ. Vessel branch quantifications were made within two retinal zones—a proximal region stretching from the optic nerve head to the radial midpoint of the retina and a distal region from the radial midpoint to the terminal edge of the tissue. For each retina, measurements were replicated at two regions of interest for each zone. The subset of vessel branches that were of interest was the terminal projections arising from parent vessels (Figure 2A). Within each region of interest (ROI), each point of vessel termination was traced back to the nearest branch node and the length measured. The distinction between parent vessel continuation and child branch formation at a node was made by looking at the angle of branching. The measured lengths were sorted into 11 µm bins (20 total) and plotted as histograms. The histogram was normalized such that the sum of bar heights was unity. Kernel density estimation (KDE) plots were also generated for each histogram. 

### 4.7. Whole Retina GFAP Density Quantification

Whole-mount retinas were labeled for GFAP and imaged as a 20× montage on a Nikon Eclipse Ni fluorescence microscope. In ImageJ, background subtraction was performed on the retinal images using a rolling ball radius of 50 pixels. A threshold was then determined that maximized the amount of fine GFAP processes visible while minimizing loss of detail that results from too low a threshold. Typical threshold percentages of the intensity profile histogram ranged from 9 to 14%. Images were subsequently binarized. In parallel, a binarized mask image was created that demarcated the region of the image with retina from background. Using a Python script, the binary GFAP image was uniformly sliced into 1000 × 1000 pixel tiles. For each tile, the ratio of area containing GFAP signal to area of white space was determined using the identically sliced binary mask image. The density measurements were logged and then mapped to a color gradient. The original binary GFAP image was then pseudo-colored with each tile showing the color corresponding to its density. Raw data were transferred to GraphPad Prism for statistical analysis.

### 4.8. Astrocyte Sholl Analysis

Images of retinas immunolabeled for GFAP were rendered binary and skeletonized using ImageJ. Sholl analyses were performed on skeletonized GFAP images using the Neuroanatomy plugin available for ImageJ. A point ROI was placed at the center of the optic nerve head to establish the starting point. The Sholl analysis graphs were produced by plotting the average number of intersects at every 100 pixels. 

### 4.9. Optomoter Response Test of Visual Acuity

The photopic visual acuity of mice was assessed by the optomotor response. Mice were placed, unrestrained, on an elevated platform centered among four adjoining LCD screens (OptoMotry; Cerebral Mechanics Inc., Lethbridge, AB, Canada). Spatial frequency thresholds were measured by assessing the oculomotor response to drifting sinusoidal gratings at 100% contrast. Grating spatial frequency (cycles/degree) was systematically adjusted based on the optomotor response noted by a naïve experimenter. Mice were tested at least twice to determine baseline spatial acuity.

### 4.10. Statistics

All data are presented as mean ± standard deviation (SD) unless otherwise stated. Graphs were made and statistical analyses were performed using GraphPad Prism version 9.0.0 (GraphPad Software, San Diego, CA, USA). We first determined whether datasets formed a normal distribution using Shapiro–Wilk tests, a test of normality appropriate for small datasets. In cases where datasets were normally distributed, we performed parametric statistics, e.g., ANOVA or *t*-tests. Where data was not normally distributed, we carried out the non-parametric version of the ANOVA, e.g., Kruskal–Wallis test. We defined statistical significance as a *p*-value of 0.05 or less. The number of measurements and specific *p*-values are indicated in results or figure legends.

## Data Availability

The data presented in this study are available on request from the corresponding author.

## Supplementary Materials

The following supporting information can be downloaded at: https://www.mdpi.com/article/10.3390/ijms23063066/s1.
