# Supplementary material for: Dysfunctional cGMP Signaling Leads to Age-Related Retinal Vascular Alterations and Astrocyte Remodeling in Mice
Source: Int J Mol Sci. 2022 Mar 12;23(6):3066. doi: 10.3390/ijms23063066 (PMC8954518; doi:10.3390/ijms23063066)
Supplement: Supplementary file 1 [file ijms-23-03066-s001.zip › ijms-1615165-supplementary.pdf]

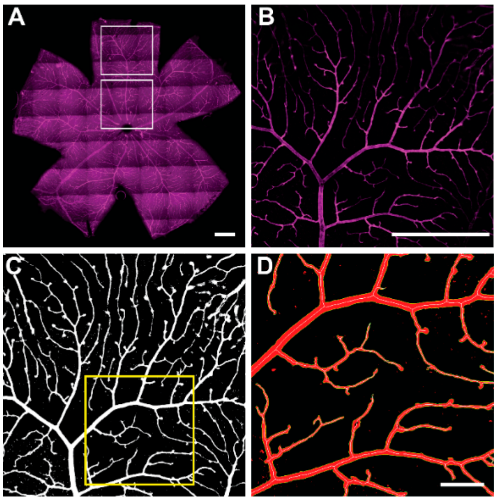

Supplemental Figure S1. REAVER Pipeline. (**A**) Representative raw fluorescence image file of whole-mount retina labeled with isolectin B4. The white boxes represent proximal and distal ROIs isolated for REAVER processing. (**B**) ROI with background subtraction. **(C)** Binarized ROI for input into REAVER. (**D**) Example REAVER segmentation output from yellow ROI in **(C)**. Segmentation outline is in yellow-green and the white segments trace the center of the vessel. Scale bars in (**A,B**) are 500 μm; in (**D**), the scale bar indicates 100 μm.

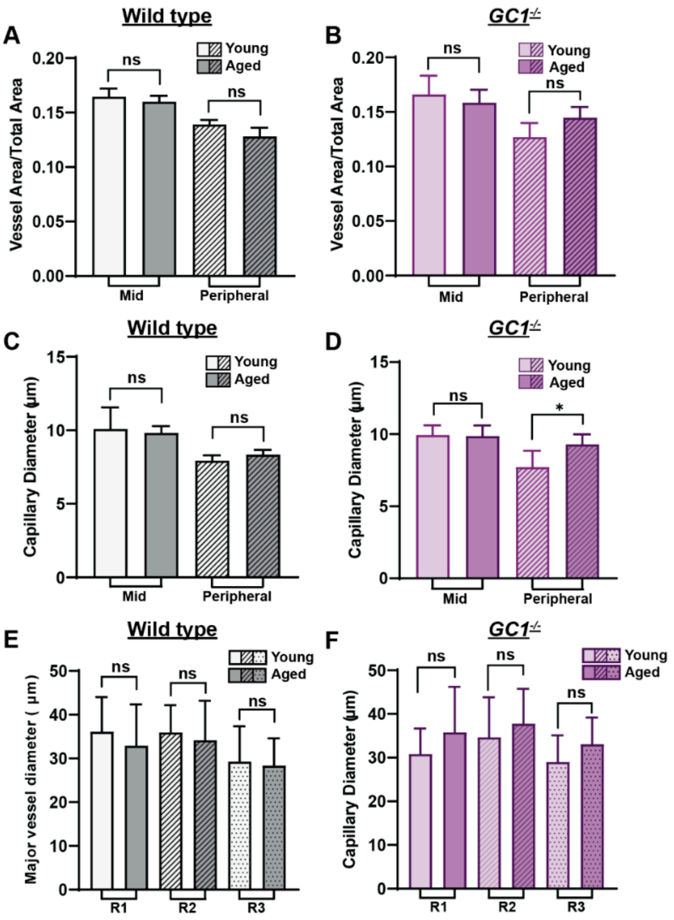

Supplemental Figure S2. Comparison of vessel phenotypes between young and aged Wt and *GC1*$^{-/-}$ mice. **(A)** No significant differences were found between the total vessel areas of young and aged Wt mice in mid (*p*=0.669) and peripheral retina (*p*=0.070). **(B)** No significant differences were found between the total vessel areas of young and aged *GC1*$^{-/-}$ mice in mid (p=718) and peripheral (p=107) retina. **(C)** No significant differences in capillary diameters were observed in young and aged Wt mice in mid (p= 0.917) and peripheral retina (p=0.784). **(D)** In *GC1*$^{-/-}$ mice, there is no significance in capillary diameter between young and old mice in mid retina (p=0.999), however, in peripheral retina, there is a progressive dilation of capillaries between young and aged mice (*p=0.011). **(E)** No significant differences in major vessels were seen between young and aged Wt animals at R1 (p=0.868), R2 (p=0.994) or R3 (p=0.993). **(F)** Similarly, in *GC1*$^{-/-}$ mice, there were no significant differences in major vessel diameters between young and old mice at R1 (p=0.388), R2 (p=0.899), or R3 (p=0.681). All data plotted as means ± S.D. Statistical analysis performed: Kruskal–Wallis one-way ANOVA.

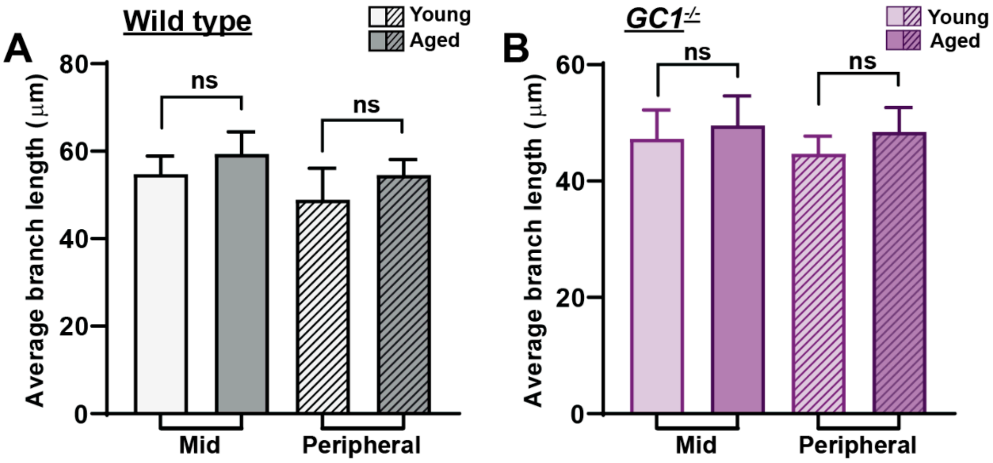

Supplemental Figure S3. Comparison of vessel branch length between young and aged Wt and *GC1*$^{-/-}$ mice. **(A)** No significant differences were found between the average branch length of young and aged Wt mice in mid (p=0.639) and peripheral retina (p=0.481). **(B)** Similarly, no significant differences were found between the average branch length of young and aged Wt mice in mid (p=0.860) and peripheral retina (p=0.611). All data plotted as means ± S.D. Statistical analysis performed: Kruskal–Wallis one-way ANOVA.
